# Are we really studying resilience in sport? A critical review of adopted methodologies

**DOI:** 10.3389/fpsyg.2023.1270887

**Published:** 2023-10-26

**Authors:** Jolan Kegelaers

**Affiliations:** ^1^Brussels University Consultation Center, Department of Psychology, Faculty of Psychology and Educational Sciences, Vrije Universiteit Brussel, Brussels, Belgium; ^2^Sport Psychology and Mental Support, Department of Movement and Sport Sciences, Faculty of Physical Education and Physiotherapy, Vrije Universiteit Brussel, Brussels, Belgium

**Keywords:** adaptation, adversity, dynamics, mental health, performance, stressors

## Abstract

Psychological resilience has gained considerable attention in sport. Nevertheless, the construct often remains poorly understood and multiple conceptual and methodological issues pervade the literature. The purpose of the present article is to provide a critical review of the commonly adopted methodologies to study resilience in sport. This review is divided into four sections. The first section will briefly discuss opposing conceptualizations of resilience as a static trait or a dynamic process. The second section will then discuss key methodological implications relating to the conceptualization of resilience as a dynamic process. In the third section, common methodologies to study resilience in sport are presented and critically reviewed. These methodologies are broadly divided into: (i) self-report resilience measures, (ii) qualitative research, and (iii) direct assessment of functioning in relation to observed adversity. In the final section, some avenues for future research are offered.

## Introduction

Resilience is commonly used to refer to the maintenance or quick recovery of functioning following stressors or adversities (Kalisch et al., 2017). This concept is particularly relevant within sport, where athletes face a wide range of potential stressors (e.g., injuries, poor performances, selection issues, interpersonal conflicts, and disease) which may impair their performance, development, and mental health (Sarkar and Fletcher, 2014; Arnold and Fletcher, 2021). Given the ubiquity of such stressors, resilience has been considered a key psychological quality for success in youth and elite sport (e.g., Rees et al., 2016; Dohme et al., 2019; Durand-Bush et al., 2022). It has been associated with both improved performance (Galli and Gonzalez, 2015) and mental health (Bryan et al., 2023) outcomes in athletes. The growing recognition for the importance of resilience in sport has also resulted in a spectacular increase of research on the topic (Bicalho et al., 2020). Nevertheless, scholars have noted that the construct often remains poorly understood (Kegelaers and Sarkar, 2021) and multiple conceptual and methodological issues pervade the literature (e.g., Sarkar and Fletcher, 2013; Galli and Gonzalez, 2015; Den Hartigh et al., 2022).

In recent years, several review papers have tried to address some of the definitional and conceptual ambiguities that exist around the construct in sport (e.g., Sarkar and Fletcher, 2014; Galli and Gonzalez, 2015; Bryan et al., 2019; Gupta and Mccarthy, 2022). Overall, synthesis of the literature provides support for the nature of resilience as a dynamic process of adaptation (Bryan et al., 2019; Gupta and Mccarthy, 2022). Resilience, in other words, emerges over time as the result of ongoing and continuously changing interactions between both individual and environmental factors (Hill et al., 2018b; Den Hartigh et al., 2022). Refining the conceptualization of resilience holds crucial implications for the methodologies used to study it. Windle (2011), for instance, argued that “how resilience is defined reflects how it might be measured and so assessment is intricately tied up with issues of definition” (p. 156). A number of papers have indeed offered methodological recommendations and guidelines for the study of resilience in sport (Sarkar and Fletcher, 2013; Galli and Gonzalez, 2015; Hill et al., 2018b; Den Hartigh et al., 2022). Despite these important conceptual and methodological advancements, no studies have critically and comprehensively reviewed whether past research is methodologically congruent with the conceptualization of resilience as a dynamic process. It remains, in other words, unclear whether the current body of work has adopted appropriate methodologies to adequately explain resilience in sport.

To address this gap in the literature, the purpose of the present article is to critically review the common methodologies adopted within resilience research in sport. According to Grant and Booth (2009), critical reviews provide “an opportunity to “take stock” and evaluate what is of value from the previous body of work” (p. 93). Critical reviews can, thus, offer a starting point for the emergence of new theoretical or analytical frameworks and methods (Grant and Booth, 2009; Snyder, 2019). The aim of the current paper is, therefore, to critically review the current evidence base through the lens of resilience as a dynamic process, as a way to spur on new research within the context of sport. Critical reviews typically do not rely on structured methodologies and a systematic search strategy, but rather aim to illustrate, synthesize, and critique key trends within the literature to let new perspectives emerge (Grant and Booth, 2009; Snyder, 2019). Hence, the aim of this article was not to provide a review of *all* resilience research in sport, but rather to provide a critical synthesis of the main methodological approaches which have been adopted within the literature. Ample references were selected to illustrate these common approaches, based on the author’s own extensive knowledge of the field as well as the resources identified within prior systematic reviews on the topic (Bryan et al., 2019; Bicalho et al., 2020; Gupta and Mccarthy, 2022). Critiques of the different methodological approaches are grounded within recent theoretical and methodological discussions surrounding the nature of resilience as a dynamic process (e.g., Sarkar and Fletcher, 2013; Galli and Gonzalez, 2015; Hill et al., 2018b; Den Hartigh et al., 2022).

This critical review will be divided into four sections. The first section will briefly discuss opposing conceptualizations of resilience as a static trait or a dynamic process. The second section will then discuss key methodological implications relating to the conceptualization of resilience as a dynamic process. In the third section, common methodologies to study resilience in sport are presented and critically reviewed. For the purpose of this review, these methodologies are broadly divided into: (i) self-report resilience measures, (ii) qualitative research, and (iii) direct assessment of functioning in relation to observed adversity. In the final section, some avenues for future research are offered. When reviewing resilience research in sport, it is important to highlight that the construct has also been applied to study the functioning of sport teams (e.g., Kegelaers et al., 2020) and organizations (e.g., Fasey et al., 2021). Although findings from this review may be relevant for the study of such collective resilience, emphasis is placed here on resilience situated at the level of the individual athlete.

## Static trait or a dynamic process?

Resilience has been defined, conceptualized, and operationalized in a myriad of ways. A central debate underpinning many of these different conceptualizations is whether it should be viewed as a static *trait* or a dynamic *process* (Fletcher and Sarkar, 2013; Métais et al., 2022). The trait perspective suggests that resilience involves an innate and dispositional capacity to respond adaptively to adversity (Connor and Davidson, 2003). This approach is widespread within the psychological literature (e.g., Wagnild and Young, 1993; Connor and Davidson, 2003; Hu et al., 2015) and has equally found its way into sport (e.g., Vitali et al., 2015; Laborde et al., 2016). For example, Vitali et al. (2015) described resilience as “a personal trait that enables an individual to thrive in the face of adversity” (p. 104). In essence, this perspective implies that resilience is a relatively stable personal quality (or constellation of personality characteristics) that is present, and therefore can be measured, at any time, even in the absence of experienced adversity.

Despite its popularity, simplicity, and potentially intuitive appeal (Kegelaers and Sarkar, 2021), the trait conceptualization has also been widely critiqued. Although a detailed discussion is beyond the scope of the current article (for more comprehensive critiques, see Luthar et al., 2000; Fletcher and Sarkar, 2013; Kalisch et al., 2017), criticisms are often grounded in the observation that resilience (a) is a contextual phenomenon (i.e., demonstrating resilience in one area of life does not necessarily mean it will be observed in another), (b) is temporally dynamic (i.e., demonstrating resilience at one point in time does not mean it will be observed at another time), (c) is influenced by environmental and situational factors, and (d) has the potential to be actively fostered or developed (Kegelaers and Sarkar, 2021). In sum, empirical observations do not seem compatible with resilience as solely a static, universal dispositional trait or personality profile.

In contrast, there now is increasing consensus both in sport (Hill et al., 2018a,b; Bryan et al., 2019; Kegelaers and Sarkar, 2021; Den Hartigh et al., 2022; Gupta and Mccarthy, 2022) and general psychology (Luthar et al., 2000; Windle, 2011; Fletcher and Sarkar, 2013; Kalisch et al., 2017; Métais et al., 2022) that resilience more accurately reflects a dynamic process of adaptation. To illustrate, Gupta and Mccarthy (2022) described resilience in sport as “the environmentally adaptable, interaction dominant, dynamic-process trajectory that encompasses a sporting individual’s metacognitive-emotional-behavioral capacities to maintain a positive equilibrium and successfully adapt to a diverse range of sport-related adversities” (p. 08). This definition underscores the temporal component of resilience, emerging as a trajectory of functioning over time in response to experienced stressors (Hill et al., 2018a). Moreover, the definition highlights the interaction dominant nature of resilience, suggesting it results from ongoing and continuously changing interactions between an individual and their environment (Hill et al., 2018b). Evidently, considering resilience as such a dynamic process, rather than a static trait, holds a number of key implications for the way it can be measured and studied. The following section will zoom in on these important methodological implications.

## Studying resilience as a dynamic process

**FIGURE 1:**
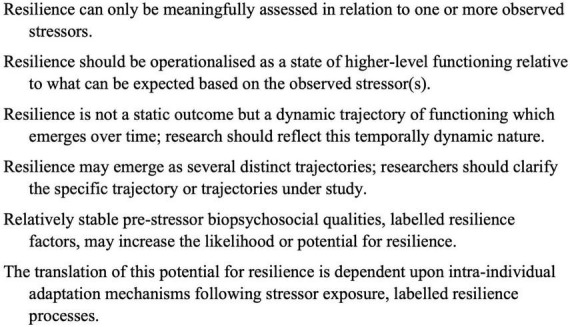
Key tenets to study the dynamic process of resilience.

Several scholars have previously discussed methodological implications for the study of resilience in sport (Sarkar and Fletcher, 2013; Galli and Gonzalez, 2015; Hill et al., 2018a,b; Den Hartigh et al., 2022). Drawing on this work, as well as the general psychology literature, these implications are summarized here as six key tenets or principles (see Figure 1). The first tenet states that resilience can only be meaningfully assessed or inferred when exposure to one or more stressors has been observed (Luthar et al., 2000; Windle, 2011; Sarkar and Fletcher, 2013; Kalisch et al., 2017). Resilience, in other words, only occurs in relation *to* something (Bonanno et al., 2015). The challenge of defining exactly what constitutes a stressor has been widely acknowledged (Fletcher and Sarkar, 2013; Kalisch et al., 2021). These can range from relatively small daily hassles to major life events (Bryan et al., 2019; Kalisch et al., 2021). Some scholars have suggested stressors are those factors statistically associated with maladjustment (Luthar and Cicchetti, 2000), whereas others have pointed out that relatively minor events, in themselves not necessarily associated with maladjustment, may over time also lead to major disruptions in functioning (Fletcher and Sarkar, 2013; Den Hartigh and Hill, 2022; Ong and Leger, 2022). For the purpose of this review, stressors are broadly defined as any type of environmental demands which have the *potential* to be appraised as threatening or harmful, and contribute to impaired functioning (Arnold and Fletcher, 2021). A crucial point of emphasis here lies in the potentiality of a stressor. Not all people will experience similar levels of stress and impaired functioning following exposure to a given stressor. In large part, resilience research is focused on understanding how and why such inter-individual differences in stress responses occur (Kalisch et al., 2021).

The second tenet states that there should be evidence of positive adaptation in the form of higher-level functioning compared to what can be expected given the experienced stressor load (Kalisch et al., 2017, 2021). Conversely, resilience losses may be observed based on functional impairment relative to the stressor load (Den Hartigh et al., 2022). Resilience should, in other words, be inferred based on individual variations of functioning in direct relation to observed stressors (Rutter, 2012). Importantly, such resilient functioning can only be meaningfully interpreted in relation to the specific context and adversity under study (Windle, 2011; Fletcher and Sarkar, 2013). To illustrate, research in developmental psychology has often considered the absence of severe psychopathology and developmental disorders in children growing up under severely adverse conditions as a sufficient criteria for resilience (Masten, 2018). In sport, on the other hand, positive adaptation is more commonly associated with athletic performance, optimal development, and mental health and well-being (Fletcher and Sarkar, 2012; Galli and Gonzalez, 2015; Bryan et al., 2023). This implies that researchers should carefully determine and justify the relevant indicators of functioning within their specific study context (Luthar et al., 2000).

The third tenet states that resilience emerges as a dynamic trajectory of functioning over time, rather than being a fixed state (Bonanno et al., 2015; Hill et al., 2018a). This means that levels of functioning change and fluctuate over time (i.e., the “dynamic” component of resilience as a dynamic process) in response to ongoing interactions between factors associated with the experienced stressors, the person, and the environment in which they function (Hill et al., 2018a,b). Importantly, such ongoing interactions do not necessarily occur linearly (Pincus and Metten, 2010; Kiefer et al., 2018). For example, Hill and Den Hartigh (2023) theorized that resilience may be eroded over time, up until a point where a relatively small perturbation can lead to a sudden and considerable drop in functioning. Crucially, research needs to account for such temporal dynamics of resilience. This requires longitudinal or temporally sensitive research designs to accurately track individuals’ trajectories of functioning and adaptation over time (Sarkar and Fletcher, 2013; Galli and Gonzalez, 2015; Hill et al., 2018a; Den Hartigh et al., 2022).

**FIGURE 2:**
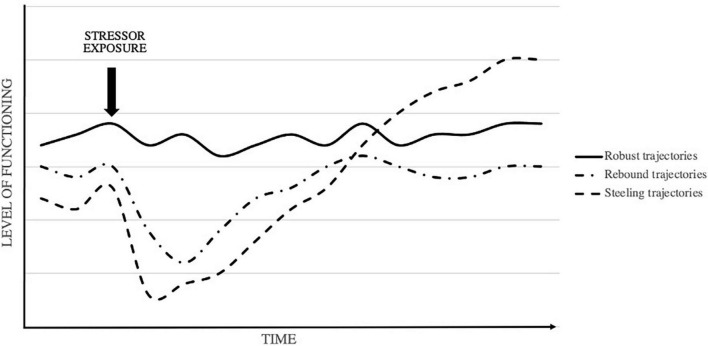
Illustration of different potential resilience trajectories.

Such temporal trajectories of resilient functioning do not emerge uniformly but can show large contextual and inter-individual variation. In this regard, researchers have noted that no single resilience trajectory exists (Bonanno and Diminich, 2013; Bonanno et al., 2015; Masten, 2018; Métais et al., 2022; Ong and Leger, 2022). In fact, multiple different types of pathways have been identified and described under a range of different labels. These can be clustered under three broad types of prototypical resilience trajectories, referred to here as *robust*, *rebound*, and *steeling* pathways (see Figure 2). Robust pathways refer to a relatively stable continued trajectory of functioning following stressor exposure (Fletcher and Sarkar, 2016; Bryan et al., 2019). Rebound pathways are characterized by a transient dip followed by relatively swift recovery to prior levels of functioning after stressor exposure (Fletcher and Sarkar, 2016; Hill et al., 2018b; Den Hartigh et al., 2022). Both robust and rebound pathways both reflect homeostatic processes of adaptation, maintaining or returning to baseline levels of functioning. However, resilience may also reflect allostatic processes of adaptation, leading to higher levels of functioning over time (Richardson, 2002; Galli and Vealey, 2008; Rutter, 2012; Seery et al., 2013). Such steeling pathways suggest that, in certain cases, exposure to stressors may ultimately lead to the acquisition and development of new resources which increase one’s resilience to future stressors (Rutter, 2012).

These different resilience trajectories reflect the heterogeneity of human adaptational processes (although some conceptual debate exists regarding which pathway constitutes the “true” nature of resilience; e.g., Den Hartigh and Hill, 2022). Nevertheless, a fundamental problems lies in the fact that the term resilience has been used indiscriminately to refer to each of these distinct pathways (Bonanno and Diminich, 2013; Den Hartigh et al., 2022). In reality, trajectories may differ substantially with regards to the temporal timeframe in which they are observed (Bonanno et al., 2015), the underlying mechanisms through which they emerge (Kiefer et al., 2018), and even the methodologies through which they can be captured (Den Hartigh and Hill, 2022; Ong and Leger, 2022). The fourth tenet, therefore, states that it is crucial for researchers to explicate the specific resilience trajectory under study and to use appropriate methodologies to operationalize and capture this trajectory over time.

In addition to measuring stressors and corresponding trajectories of functioning, the fifth tenet states that resilience research should also assess the determinants for positive adaptation (Windle, 2011; Sarkar and Fletcher, 2013). Such *resilience factors* are commonly referred to using a range of different labels, including protective factors, promotive factors, resources, or assets (Windle, 2011). For the purpose of this review, resilience factors are broadly considered as the pre-stressor qualities that increase the likelihood or potential of an individual being able to withstand or quickly recover from a stressor (Kalisch et al., 2017). These factors can be identified at multiple levels of influence, including the individual, the close social environment, and even the wider community or society (Windle, 2011; Métais et al., 2022). Gupta and Mccarthy (2022) refer to this assortment of different resilience factors as an individual’s “biopsychosocial protective filter” (p. 11). A range of potential determinants, primarily situated at the individual level, have already been proposed in sport. These include constructs such as self-efficacy, motivation, optimism, positive personality, mental toughness, self-awareness, and perceived social support (Sarkar and Fletcher, 2014; Bryan et al., 2019; Gupta and Mccarthy, 2022).

Although resilience factors represent a pre-stressor potential for positive adaptation, translating this potential into actual resilient outcomes is not always a given. Despite over 40°years of research in wider psychology, there currently exists no unified set of universally applicable resilience factors. Most identified resilience factors only explain a small portion of the variance in observed functioning (Kalisch et al., 2017). In fact, in certain contexts commonly accepted resilience factors (e.g., social support) may even hamper resilience (Mummery et al., 2004; Hill et al., 2018b). Moreover, resilience factors have been typically presented as population-wide protective qualities, identified through qualitative research or group-level statistics. However, such group-level factors provide limited insights into the way specific individuals adapt to their particular circumstances (Hill et al., 2021). The final tenet, therefore, states that research should also assess the *resilience processes* through which individuals translate their potential for positive adaptation (i.e., available resilience factors) into actual resilient outcomes. Such resilience processes represent the intra-individual cognitive, emotional, and behavioral response mechanisms that moderate the relationship between available resilience factors and adaptive responses to specific stressors (Kalisch et al., 2017; IJntema et al., 2019; Infurna, 2020). Kalisch et al. (2017) speculated that underpinning the broad range of potential resilience factors may in fact be a limited number of resilience processes producing actual adaptive outcomes. For instance, cognitive responses such as stressor reappraisal (Riepenhausen et al., 2022) or self-reflection (Crane et al., 2019) have been implied as key resilience processes. Appreciating the distinction between resilience factors and processes illustrates that both *nomothetic* (group-level patterns and generalizations) and *idiographic* (intra-individual processes and changes) methods are needed to capture how athletes manage to maintain or reach positive levels of functioning following stressor exposure (Den Hartigh et al., 2022; Ong and Leger, 2022). Nomothetic approaches can provide insight into the broad group-level resilience factors that may increase the likelihood of an individual demonstrating resilience. However, idiographic measures are needed to understand the intra-individual processes through which the individual translates their potential into actual resilience.

## Current methodologies to study resilience in sport

Building on the key tenets presented in the previous section, we can now start to critically examine the common methodologies used to study resilience in sport. For the purpose of this review, three broad methodologies will be distinguished: (i) quantitative self-report measures, (ii) qualitative research, and (iii) direct assessment of functioning in relation to adversity. The goal of critically discussing these different methodologies is not to call out specific studies or researchers. Rather, it is meant to examine if and to which extent the current research base is methodologically congruent with the conceptualization of resilience as a dynamic process, that is to which extent are they aligned with and consistent with the key principles discussed in the previous section, and highlight key areas for improvement in future research.

### Quantitative self-report measures

The predominant approach to measure resilience in sport is through the use of existing quantitative self-report resilience scales (e.g., Hosseini and Besharat, 2010; Belem et al., 2014; Cowden et al., 2016b; Secades et al., 2016; Drew and Matthews, 2019; Moen et al., 2019; Madsen et al., 2021; Zhang et al., 2023). In their review, Bicalho et al. (2022) identified 11 different scales which have been used in sport. These include common measures such as the Connor-Davidson Resilience Scale (CD-RISC; Connor and Davidson, 2003), the Resilience Scale (RS; Wagnild and Young, 1993), the Brief Resilience Scale (BRS; Smith et al., 2008), and the Resilience Scale for Adults (RSA; Friborg et al., 2003). Reviews examining the psychometric qualities of these existing self-report measures indicate that, overall, their quality is moderate and currently no “gold standard” exists (Windle et al., 2011; Pangallo et al., 2015). Moreover, these instruments were originally developed for use in other populations (e.g., clinical patients) and remain largely untested in sport (Bicalho et al., 2022). To date, only the psychometric properties of the CD-RISC (Gucciardi et al., 2011; Gonzalez et al., 2016) and the RSA (Cowden et al., 2016a) have been studied in athlete populations. Nevertheless, the contextual appropriateness of these measures remains questionable and scholars have advocated for the development of a novel sport-specific resilience scale (Gucciardi et al., 2011; Sarkar and Fletcher, 2013; Galli and Gonzalez, 2015; Gonzalez et al., 2016; Wagstaff et al., 2017; Bicalho et al., 2022; Gupta and Mccarthy, 2022; Zhang et al., 2023). Some authors have, indeed, attempted to develop such sport-specific measures (e.g., Subhan and Ijaz, 2012; Ueno and Shimizu, 2012), although the uptake of these scales remains sparse.

Despite the call for a new sport-specific instrument being widely shared, some important issues underpinning the use of self-report resilience measures have remained largely unaddressed within sport. Starting with the most fundamental issue, the existing self-report resilience scales do not capture temporally dynamic trajectories of functioning in direct relation to observed stressor experiences (Sarkar and Fletcher, 2013). As such, “resilience measures” paradoxically do not measure actual resilience (Bonanno et al., 2015; Kalisch et al., 2017; Ong and Leger, 2022). If self-report scales do not assess resilience itself, then what do they measure? In this regard, two broad approaches can be distinguished. The first approach is represented in the BRS (Smith et al., 2008), which aims to assess an individual’s *perceived ability* to adapt to stressful experiences (i.e., demonstrate resilience). The items within this instrument refer toward a general ability to bounce back from stressors (e.g., “*It does not take me long to recover from stressful events*”), indicative of rebound resilience trajectories (Den Hartigh and Hill, 2022). However, such an assessment of perceived ability is still prone to errors in self-perception and reporting bias and does not account for the contextual specificity and complexity of resilience. As such, the BRS should, at best, only be considered as a proxy measure for resilience.

The second, more common, approach is for existing self-reports scales to (explicitly or implicitly) measure different resilience factors (Windle et al., 2011; Kalisch et al., 2017). That is, rather than assessing actual adaptation processes, these scales aim to measure supposed determinants for adaptation. Several additional challenges are related to such an approach. First, this creates a circularity issue, whereby many scholars blur the distinction between antecedents (i.e., resilience factors) and the outcome of resilience (Sarkar and Fletcher, 2013). Second, most of these scales are ontologically conflicting with a dynamic process view of resilience, since they were developed from an explicit trait-based conceptualization (e.g., Wagnild and Young, 1993; Connor and Davidson, 2003). Third, in line with their trait-based conceptualization, most scales focus on measuring innate personal resilience factors. As such, they fail to account for the crucial dynamic interactions between an individual and their environment in the process of adaptation and neglect situational or environmental resilience factors (Windle et al., 2011; Pangallo et al., 2015; Wagstaff et al., 2017). It should be mentioned, though, that some notable exceptions (i.e., scales which also attempt to measure environmental resilience factors) have started to be used sporadically in sport (e.g., Cowden et al., 2016a; Zhang et al., 2023). Fourth, there often is little or questionable theoretical justification provided for the inclusion of different items within these resilience scales (Windle et al., 2011; Sarkar and Fletcher, 2013; Pangallo et al., 2015). As such, it remains unclear what the predictive value is of the included resilience factors for actual observed resilience. In this regard, research outside of sport has demonstrated that self-report scales, such as the CD-RISC, hold limited potential to predict longitudinal trajectories of resilient functioning (e.g., van der Meulen et al., 2018; Booth et al., 2022).

Another challenge related to the use of self-report scales is that these typically reflect a nomothetic approach to measuring resilience factors. Group-level statistics are typically used to discern common patterns of resilience factors for the population as a whole (Hill et al., 2018b). However, Hill et al. (2021) demonstrated that group-level resilience factors are poor predictors for individual trajectories of resilience. Hence, the current self-report resilience measures hold little value to understand idiographic adaptation processes underpinning resilience (Hill et al., 2021). Finally, self-report scales fail to capture the temporal dynamics of resilience (Pangallo et al., 2015). Although not a limitation of these scales *per se*, quantitative research has overly relied on cross-sectional designs to study resilience in sport. Such snapshot assessments do not account for the temporal dimension of resilience and fail to consistently and accurately capture different resilience pathways (Galli and Gonzalez, 2015; Hill et al., 2018b; Den Hartigh et al., 2022). However, even when used in longitudinal designs, self-report scales do not capture the temporally dynamic nature of resilience. To illustrate, the limited available longitudinal studies in sport have rather focused on correlations between static resilience scores across different points of a season (Secades et al., 2016; Barczak-Scarboro et al., 2022) or using one-off resilience scores to predict changes in other outcome measures over time (Ueno and Suzuki, 2016; Sorkkila et al., 2019). As such, these studies fail to provide insight into different pathways (Galli and Gonzalez, 2015; Pangallo et al., 2015) and capture the temporally dynamic adaptation process that lies at the heart of resilience (Cosco et al., 2017; Hill et al., 2018a).

In sum, although widely used in sport, the currently available quantitative self-report resilience measures seem ill-suited to measure the temporally dynamic and interaction dominant process of resilience (Kalisch et al., 2017).

### Qualitative research

Several seminal resilience studies in sport have adopted a qualitative design, particularly through the use of semi-structured interviews (e.g., Galli and Vealey, 2008; Fletcher and Sarkar, 2012). Qualitative research can provide important contributions to the study of resilience in several ways. It can uncover “unnamed processes” (Ungar, 2003) and mechanisms (Infurna, 2020) that remain obscured within quantitative approaches. Qualitative designs are suited for both nomothetic and idiographic approaches. Methods such as grounded theory (Holt, 2016) can help identify population-level resilience factors and generate novel theoretical frameworks (Fletcher and Sarkar, 2012; Galli and Gonzalez, 2015). On the other hand, phenomenological (Smith, 2016) or narrative (Smith and Sparkes, 2009) approaches may provide insights into intra-individual lived experiences and meaning-making processes (Galli and Gonzalez, 2015; Infurna, 2020). To illustrate, Smith (2013) explored how male disability athletes’ personal narratives of “being resilient” could be a resource for health and well-being, but can equally become a barrier for health-promoting behaviors when embedded within perceived virtues of “toughness” or “not caring too much about health.” Finally, qualitative methods can enrich quantitative findings as part of mixed-methods designs (Ungar, 2003; Wagstaff et al., 2017; Infurna, 2020). For example, qualitative research can inform theory development for subsequent quantitative work or, alternatively, validate and explain quantitative findings by matching these with meaningful fluctuations in individual lived experiences (Windle et al., 2011).

Although qualitative research has the potential to provide important insights, some challenges need to be highlighted. Qualitative studies rely on purposefully sampling participants who putatively demonstrated resilience in the past. However, in many studies it remains ambiguous whether the selected sample is indeed appropriate to study the construct of resilience. In other words, authors often fail to clarify if and to which extent their participants did indeed demonstrate positive functioning in direct relation to specific experienced stressors. Regarding experienced stressors, some qualitative studies focused on resilience to one clear adversity, including physical disability (Machida et al., 2013; Smith, 2013), performance slumps (Brown et al., 2020), terrorism (Timm et al., 2017), or the COVID-19 pandemic (Gupta and McCarthy, 2021; Johnson et al., 2022). Other studies included a much broader range of stressors (Galli and Vealey, 2008; Fletcher and Sarkar, 2012; Brown et al., 2015; Kegelaers and Wylleman, 2019; Sarkar and Hilton, 2020). Galli and Gonzalez (2015) pointed out that studying such a heterogenous group of stressors may preclude deeper insight into adaptation processes to specific types of stressors. Moreover, some papers altogether failed to describe the stressors in relation to which they studied resilience. For example, White and Bennie (2015) simply referred to “challenges experienced in gymnastics” (p. 383), without further specification.

More problematically, most qualitative studies fail to clearly demonstrate positive functioning in relation to the reported stressors and rather use indirect approaches to infer resilience. One common approach is to infer positive functioning based on participants’ overall level of performance (e.g., Fletcher and Sarkar, 2012; Brown et al., 2020; Sarkar and Hilton, 2020). For example, Fletcher and Sarkar (2012) studied the experiences of Olympic champions, arguing that they “have been shown to possess certain psychological characteristics that enable them to withstand stressors and that set them apart from less successful athletes” (p. 670). However, the level of performance at the time of the study does not necessarily tell us anything about how participants adapted to specific stressors in the past. Performance is complex and multifaceted, with many other factors outside of resilience potentially contributing to one’s level of performance (Rees et al., 2016). This also raises the question whether the described findings are particularly relevant to resilience or whether they might reflect broader psychological characteristics underpinning exceptional performance. Indeed, Fletcher and Sarkar (2012) identified several characteristics (e.g., motivation, confidence, and focus) which are often considered crucial psychological factors underpinning sporting performance, independent from resilience (e.g., Dohme et al., 2019; Durand-Bush et al., 2022).

Another common approach to infer resilience is using referrals from important others (e.g., coaches and high-performance directors) (Galli and Vealey, 2008; Brown et al., 2015; Kegelaers and Wylleman, 2019). Relying on such subjective external evaluations equally has its evident challenges. Researchers have highlighted that colloquial understandings of resilience often differ substantially from its meaning as a scientific construct (Bryan et al., 2019; Kegelaers and Sarkar, 2021). As such, relying on external judgments might be biased by some of the common popular misunderstandings that exist around the construct.

Finally, self-report resilience scales have also been used to purposefully sample participants (Gupta and McCarthy, 2021). In addition to the multiple problems related to self-report scales highlighted earlier, such an approach uses essentially arbitrary cut-off points to assess whether someone should be considered resilient or not. Overall, self-report scales lack the theoretical support, validity, and normative data to effectively guide purposeful sampling procedures for qualitative research.

In addition to challenges related to purposeful sampling, qualitative studies have also been critiqued for their overreliance on cross-sectional retrospective designs (Galli and Gonzalez, 2015). The use of retrospective designs may pose a risk for recall bias, particularly when there is a long time between the demonstration of resilience and the actual interviews (Galli and Gonzalez, 2015; Wagstaff et al., 2017). To illustrate, some participants within the study of Fletcher and Sarkar (2012) were asked about the resilience they exhibited in relation to winning an Olympic medal as much as 40°years prior to the interviews. One-off interviews may also provide limited insights into the temporal dimension of resilience (Galli and Gonzalez, 2015). Although some notable qualitative studies have tried to address the temporal process of resilience, particularly through the use of more time-sensitive narrative approaches (Galli and Vealey, 2008; Gupta and McCarthy, 2021), most qualitative studies have focused solely on identifying resilience factors without exploring how such qualities are deployed over time. Moreover, most qualitative studies failed to clarify which specific resilience pathways they examined. In order to account for such limitations, scholars have increasingly started to advocate for longitudinal qualitative designs to develop a better understanding of how the process of resilience unfolds over time (Sarkar and Hilton, 2020; Johnson et al., 2022).

A final important limitation of qualitative resilience research is the potential for survivorship bias (Uphill and Hemmings, 2017). Survivorship bias is defined as “a logical error of concentrating on the people or things that made it past some selection process and overlooking those that did not” (Lockwood, 2021, p. 2). By purposefully sampling only those individuals who putatively demonstrated resilience, important insights derived from looking at those who did not reach the same level of adaptation might be missed. Indeed, Uphill and Hemmings (2017) argued that “turning our attention to those athletes who perhaps do not make the pinnacle of their sport and where they are vulnerable to being “hit” may provide the practitioner with enhanced understanding of how to mitigate against such risks” (p. 303). As such, failing to consider alternative lived experiences may lead to incomplete or distorted conclusions regarding the factors that can distinguish resilient and non-resilient responses.

In sum, although qualitative inquiry holds potential to advance the study of resilience, research can be improved by ensuring adequate purposeful sampling, accounting for temporal dynamics, and avoiding survivorship bias.

### Direct assessment of functioning in relation to adversity

The final common methodology has tried to directly associate objective indicators of functioning to experienced adversity, through a series of field and experimental studies. Most of these studies considered performance as the primary indicator of resilient functioning. In their field study, Mummery et al. (2004) conceptualized resilience as a successful performance (i.e., personal best) following initial performance failure during national swimming championships. Other scholars have adopted an experimental approach to manipulate adversity exposure by providing failure feedback following a sporting task (Seligman et al., 1990; Martin-Krumm et al., 2003; Gonzalez et al., 2018; Green et al., 2018). For example, Seligman et al. (1990) falsely informed competitive swimmers that their time on an initial swimming trial was slower than their actual swim times and used performance improvements on a subsequent trial as a marker for resilience. Finally, Jones and Jetten (2011) examined physiological responses to physical challenges as markers of resilience. In two separate experiments, resilience was conceptualized as faster heart rate recovery (study 1) and greater endurance (study 2) following novel physical stressors.

These studies are aligned with the first two tenets to study resilience as a dynamic process, as they include both a specific stressor and a corresponding measure of positive functioning (Sarkar and Fletcher, 2013). Nevertheless, several limitations still need to be highlighted. First, many of these experimental studies have relied on novice athlete populations in the form of university students (Mummery et al., 2004; Jones and Jetten, 2011; Green et al., 2018). Although this choice may be understandable from a practical perspective, it remains unclear to which extent these findings are generalizable to actual high-performance athletes (Green et al., 2018). Second, these studies focus on resilience in response to a singular stressor. In reality, stressors often do not occur in isolation. Rather, people may experience multiple simultaneous stressors, situated within different life domains, which reciprocally influence each other (Infurna, 2020). Hence, it is ambiguous whether findings from these studies transfer to different types of stressors or when multiple simultaneous stressors are present (Sarkar and Fletcher, 2013; Galli and Gonzalez, 2015). Moreover, it remains unclear whether the studied stressors were even perceived as actual stressors by the study participants (Galli and Gonzalez, 2015; Wagstaff et al., 2017). For example, in the study of Mummery et al. (2004), it may well be that participants made a conscious choice to conserve energy during early trials, and therefore did not consider the initial performance failure as a stressor.

Third, with one exception (Jones and Jetten, 2011), all studies considered performance as the key indicator of positive functioning following stressor exposure. Several scholars have argued that positive functioning should, ideally, be assessed across multiple domains (Luthar et al., 2000; Sarkar and Fletcher, 2013; Infurna, 2020). Positive functioning in one domain does not necessarily mean that an individual will equally demonstrate such functioning in other domains as well (Luthar et al., 2000). As such, drawing strong inferences based on functioning in a single domain may lead to spurious and premature conclusions about the nature of resilience. Moreover, it can be questioned to which extent performance would even be the most appropriate indicator of resilience within sport. As highlighted, there are many conceivable reasons why an athlete does not reach a certain performance level (e.g., opponents and performance conditions), even when they have adapted well to experienced stressors. As such, other proximal indicators of functioning may be equally relevant to assess athletes’ resilience, including effort, affect, or well-being (Galli and Gonzalez, 2015; Den Hartigh et al., 2022).

Finally, these studies assessing functioning in direct relation to an experienced stressor still fail to account for the temporal aspects of the resilience process. They may, in other words, demonstrate a resilient outcome, but they don’t examine the process through which such an outcome is obtained. As highlighted by Den Hartigh and Hill (2022) measuring functioning at one time point following a stressor does not capture resilience fluctuations or provide insights into the trajectories leading to such outcomes.

In sum, although these studies examine resilience in direct relation to an observed stressor, important questions can still be raised regarding their ecological validity and ability to explain the complexities and temporal aspects of the resilience process in relation to real-life stressors.

## Avenues for future research

We can now start considering how future sport-specific research may be advanced to align more closely with the nature of resilience as a dynamic process. In this final section, several specific avenues and considerations for future research are presented. To be clear, the aim here is to offer a range of potentially interesting opportunities rather than advocating for a single approach. Moreover, in-depth discussions of specific novel methodologies to study resilience are beyond the scope of this article and can be found elsewhere (e.g., Kalisch et al., 2021; Den Hartigh et al., 2022). It is hoped that this section may provide inspiration for researchers to critically reflect on their own methodologies to study resilience in sport in the future.

It is widely accepted that prospective longitudinal research designs are crucial to advance the study of resilience as a dynamic process (Sarkar and Fletcher, 2013; Bonanno et al., 2015; Cosco et al., 2017; Hill et al., 2018b). Such prospective longitudinal designs involve the ongoing monitoring of both stressor exposure and corresponding functioning. With regards to stressor exposure, researchers are advised to adopt a multilevel approach and consider a broad range of psychological and physiological stressors within the sporting environment (Sarkar and Fletcher, 2014; Arnold and Fletcher, 2021; Den Hartigh et al., 2022). Researchers should then carefully consider which variables are considered contextually relevant markers for corresponding levels of functioning. Monitoring athletes’ functioning may be based on self-report measures to assess, for example, subjective well-being (Bryan et al., 2023) or satisfaction across different life domains (Wylleman and Rosier, 2016). Ideally, however, such measures would be complemented with real-world indicators of observable functioning (Andersen et al., 2007). Performance outcomes remain a logical and relevant indicator of functioning within sport environment (e.g., Meggs et al., 2015). However, given the complexity of predicting and explaining performance highlighted earlier, researchers may also consider parameters such as physiological data or observable (e.g., on pitch) behaviors (Den Hartigh et al., 2022). An important consideration for monitoring stressors and functioning over time is the appropriate frequency and duration of the adopted time windows. Frequent measurement points (i.e., beyond simple two- or three-way wave measurements) are needed to capture non-linear trajectories of resilient functioning (Cosco et al., 2017). Moreover, depending on the context and the experienced stressor, some resilience processes may emerge over a period of months whereas others emerge over a matter of days or even hours. This necessitates proper measurement infrastructure to allow for sufficient structured measurements points, which are frequent enough to capture the proposed adaptation process (Den Hartigh et al., 2022). In this regard, ecological momentary assessments may be particularly valuable to frequently track both stressors and functioning over a prolonged period of time (Ong and Leger, 2022).

Researchers should also carefully consider how different resilience trajectories are appropriately operationalized and quantified within prospective longitudinal designs. In recent years, methodological advances have been offered which may allow for ecological, person-centered assessments of different resilience trajectories. For instance, scholars have advocated for an area under the curve (AUC) approach to quantify resilience within intensive time series (Den Hartigh et al., 2022; Baretta et al., 2023). Such an approach is particularly suited to quantify the effectiveness with which an individual is able to return to previous levels of functioning (i.e., rebound resilience). This AUC approach may then be able to detect instances of “critical slowing down,” predicting major episodes of resilience loss (Den Hartigh et al., 2022). Within general psychology, a residualization approach has also increasingly been adopted to quantify resilience (e.g., Amstadter et al., 2014; Booth et al., 2022). This approach uses individual residual scores of the normative relationship between stressor load and functioning (Kalisch et al., 2021). Residual scores, thus, offer a measure of stress reactivity, whereby resilience is quantified as better-than-expected functioning relative to the experienced stressor load (Kalisch et al., 2021). This residualization approach may be used to quantify robust resilience trajectories over relatively long time windows (Chmitorz et al., 2021) as well as across day-to-day responses to experienced stressors (Wackerhagen et al., 2023).

Qualitative research equally holds strong potential to further advance our understanding of resilience in sport (Ungar, 2003; Galli and Gonzalez, 2015; Infurna, 2020). However, future research should adopt careful sampling criteria to ensure participants have actually demonstrated resilience. To this end, researchers should establish and outline the specific stressors in relation to which resilience is studied. Moreover, researchers should establish observable and contextually relevant indicators of positive functioning, rather than inferring resilience based on secondary outcomes such as judgments of others (e.g., Galli and Vealey, 2008) or the use of self-report measures (e.g., Gupta and McCarthy, 2021). One potential way to purposefully sample participants based on observed trajectories of functioning is to integrate qualitative methods within prospective longitudinal designs (Infurna, 2020). Complementing longitudinal designs with qualitative work may provide insight into the idiographic lived experiences of individuals having observably demonstrated resilience. Sampling individuals with observable patterns of functioning may also allow for comparisons of different resilience trajectories and explore meaningful differences in lived experiences between groups. Moreover, contrasting different patterns of functioning would allow researchers to compare experiences of individuals who demonstrated resilience to those who did not, thereby mitigating the risk for survivorship bias (Uphill and Hemmings, 2017) and develop a more nuanced understanding of the intersection between resilience and vulnerability. When conducting qualitative research, it remains crucial to account for the temporal component of resilience. This may be achieved through the incorporation of timelining, which allows researchers and participants to draw out temporal components of personal narratives and experiences (Sheridan et al., 2011). However, ideally qualitative studies would also collect data longitudinally to capture temporal aspects of resilience *in situ*. To this end, complementing sporadic interviews with regular (e.g., daily) or event-contingent (e.g., following specific stressors) written diary entries may be a particularly promising research avenue (Day, 2016).

Ultimately, developing a better understanding of resilience should lead to the design and evaluation of novel interventions (Galli and Gonzalez, 2015; Kegelaers, 2019). To date, intervention research in sport remains notably sparse. Moreover, the limited existing intervention studies (see Deen et al., 2017; Chandler et al., 2020; Kegelaers et al., 2021; Vella et al., 2021) suffer from many of the same limitations as the broader literature. For example, all interventions have used self-report resilience measures (e.g., CD-RISC) as a surrogate to evaluate intervention outcomes in the absence of actual observed stressor exposure. Given the ample limitations of existing resilience scales, Windle et al. (2011) highlighted that it remains doubtful whether these are sensitive to capture intervention effects. Moreover, the design of these interventions has been inconsistent with a dynamic process view of resilience. Most studies have adopted a straightforward pre-post design, which fails to capture temporal processes or gain insight into different resilience trajectories (Chmitorz et al., 2018; IJntema et al., 2019). These shortcomings limit the ability to infer the actual effectiveness of these interventions. Future intervention research is therefore needed, which carefully aligns its conceptualization, design, and methods with contemporary process conceptualizations of resilience (Chmitorz et al., 2018; IJntema et al., 2019). Such interventions may aim to develop resilience in response to both “naturally” occurring or simulated stressors, in the form of carefully planned disruptions (Kegelaers and Oudejans, 2022). Longitudinal follow-up assessments of relevant markers of functioning can then capture dynamic changes in individuals’ adaptation to these stressors over time and compare these with relevant control populations (e.g., athletes not receiving the intervention) (Chmitorz et al., 2018).

## Concluding remarks

The aim of this article was to critically review and discuss the commonly adopted methodologies to study resilience in sport, in light of the conceptualization of resilience as a dynamic process. It should be acknowledged that defining resilience, and consequently determining appropriate methodologies, is fundamentally an ontological issue. For instance, scholars conceptualizing resilience as a stable personality trait rather than a dynamic process may consider many of the discussed methodologies entirely appropriate. However, it is clear from recent review studies (Hill et al., 2018b; Bryan et al., 2019; Den Hartigh et al., 2022; Gupta and Mccarthy, 2022) that the dynamic process perspective is increasingly becoming the dominant theoretical lens to study resilience in sport. Given this growing consensus, it is remarkable that the current evidence base remains severely hampered by research practices which are largely incongruent with its nature as a dynamic process. Moreover, this incongruence is evident across different types of methodologies. It is, therefore, hoped that this critical review may provide an impetus for a new wave of resilience research in sport. Evidently, designing and conducting research which is compatible with the dynamical process of resilience (e.g., prospective longitudinal research) can be challenging and resource intensive, especially within a small and volatile domain such as sport. At the same time, sport offers a “natural laboratory” to study how individuals respond to adversity (Sarkar and Fletcher, 2014). Athletes are unique in the way they deliberately and voluntarily expose themselves to a wide range of stressors on an almost daily basis. The frequency and consistency in which stressors occur allows for meaningful prospective assessments of resilience, which may not always be feasible in other domains. As such, sport is well-suited to advance resilience methodologies in a way that is scalable to other fields (Den Hartigh et al., 2022).

## Author contributions

JK: Conceptualization, Writing – original draft.
